# Elaboration of the Demulsification Process of W/O Emulsion with Three-Dimensional Electric Spiral Plate-Type Microchannel

**DOI:** 10.3390/mi10110751

**Published:** 2019-11-01

**Authors:** Zhengdong Ma, Yadong Pu, Diliyaer Hamiti, Meixiu Wei, Xiao Chen

**Affiliations:** 1 Lab of Microfluidic Synthesis & Separation, College of Chemistry & Environment Protection Engineering, Southwest Minzu University, Chengdu 610041, China; mazhengdong18@gmail.com (Z.M.); puyadong28@gmail.com (Y.P.); diliyar0513@gmail.com (D.H.); chemmeixiu@gmail.com (M.W.); 2 School of Chemical and Biomolecular Engineering, Georgia Institute of Technology, Atlanta, GA 30332, USA

**Keywords:** microchannel, demulsification, induction period, residence time, cut size

## Abstract

Rapid and efficient demulsification (destabilizing of an emulsion) processes of a water in oil (W/O) emulsion were carried out in a three-dimensional electric spiral plate-type microchannel (3D-ESPM). In this experiment, the demulsifying efficiency of emulsions by 3D-ESPM was compared with that by gravity settling, the factors influencing demulsifying efficiency were investigated, and the induction period, cut size and residence time in the demulsification process were studied. The results showed that in contrast to the gravity settling method, 3D-ESPM can directly separate the disperse phase (water) instead of the continuous phase (oil). The maximum demulsifying efficiency of W/O emulsion in a single pass through the 3D-ESPM reached 90.3%, with a microchannel height of 200 μm, electric field intensity of 250 V /cm, microchannel angle of 180°, microchannel with 18 plates and a flow rate of 2 mL /min. An induction period of 0.6 s during the demulsification process was simulated with experimental data fitting. When the residence time of emulsion in 3D-ESPM was longer than the induction period, its demulsifying efficiency increased as the increase of the flow velocity due to the droplet coalescence effects of Dean vortices in the spiral microchannel. For this device a cut size of droplets of 4.5 μm was deduced. Our results showed that the demulsification process of W/O emulsion was intensified by 3D-ESPM based on the coupling effect between electric field-induced droplets migration and microfluidic hydrodynamic trapping.

## 1. Introduction

Emulsions are widely used and play a vital role in industrial processes, agricultural industry and daily life. However, used emulsions are usually an environmental hazard [1], such as drilling fluid, microemulsion cutting fluid and pesticides solutions, which cannot be discharged directly into the environment before demulsification or recovering oil and water in emulsions [2]. Traditional techniques used for demulsifying emulsions were usually divided into two major types: Physical methods and chemical methods; the former utilize physical forces like gravity, centrifugal force [3,4], electrostatic interaction [5,6,7], thermal treatment [8] and ultrasonic [9,10] to destabilize and demulsify emulsions. In addition, the latter type involved implementing demulsification by adding chemical demulsifiers [11]. However, for most of these methods, disadvantages of tedious processing, secondary pollution and high cost were reported recently.

This was especially the case when demulsifying water in oil (W/O) emulsions with micrometer-sized droplets; the traditional technique was complained of low efficiency or no effect, and new methods of microwave heating [12,13] and microbiological [14] were resorted to.

Nowadays, electric demulsification, commonly known as electrocoalescence, is usually used to separate W/O emulsions in industry. The technology of electrocoalescence employs electrostatic forces to separate water droplets in the W/O emulsions without any chemical additions, and can be used conveniently for large-scale industrial production, and has evolved as the most efficient and economical way for demulsifying W/O emulsions [15]. However, electric demulsification in the industry usually requires a high electric field strength of 0.83–10 kV/cm and ultrahigh voltage (6–12 kV), therefore a special design of the electrostatic coalescer is necessary to avoid insulation breakdown and short current. Moreover, the interaction of electrodes with emulsions may be involved in the process, which might lead to some electrochemical reactions. Accordingly, we combined the electric field with microchannel technology to demulsify W/O emulsions in order to greatly reduce the voltage demand. Our group had fabricated a three-dimensional electric spiral plate-type microchannel (3D-ESPM) to separate water in kerosene (W/O) emulsions using the double coupling actions of a direct current (DC) electric field and special flow in the spiral microchannel [16]. The results revealed that the dual coupling of this DC electric field and spiral microchannel within the 3D-ESPM can demulsify the water in kerosene emulsions with high efficiency.

Since 2004, plate-type microchannels were used to for the demulsification of O/W emulsions, and these reached very high demulsifying efficiencies through the surface force among oil droplets and asymmetric hydrophobic/hydrophilic walls. Okubo applied a 10 mm-in-length glass- Polytetrafluoroethylene (PTFE) microchannel to investigate the separation of the oil and water mixture and achieved almost 100% liquid-liquid separation in 0.01 s [17]. Kolehmainen fabricated a 200 mm-in-length PTFE-SS (Stainless Steel) microchannel device and achieved almost 100% separation efficiency for ShellSol (an isoparaffin synthetic hydrocarbon solvent) in the water mixture [18]. Xiao Chen designed and manufactured a plate-type PTFE-SS microchannel to achieve the continuous demulsification for kerosene in water emulsions with emulsifier, and the demulsification process was repeated 5 to 20 times, and its demulsification efficiency can be up to 90% [19]. All the above papers pointed out that the process intensification of microchannels on droplets coalescence was due to the special flow pattern [17,18,19,20] produced by O/W emulsions flowing through the microchannel, like oil droplets absorbed on the hydrophobic wall [17,19], the wall slippage [19,21,22] and the confined flow [19], which can generate an instability of O/W emulsions, accelerate the coalescence of oil droplets and result in phase separation.

Droplet coalescence is divided into passive droplet coalescence and active droplet coalescence [23]. The passive method is mainly implemented by changing the structure of the device. The active method is mainly implemented by using an applied electric field, a sound field, or the like [24,25,26,27]. Some researchers [16,28] employed both methods together to get a better separation efficiency. John S. Eow proposed that the droplet coalescence process can be described in three stages: Droplets approaching each other, the process of film thinning/drainage and film rupture leading to droplet-droplet coalescence [5]. Droplets approaching each other and the process of film thinning/drainage are understood as the preparatory stages of the coalescence process, which takes a short time to lead to the consequent obvious demulsification phenomena, and this time would be considered as the induction period before demulsification.

Due to the unique micro-scale characteristics of microchannels, the manipulation of particles has become more flexible and controllable. The broadening and converging microchannels are commonly used microstructures for getting droplets close each other and appearing as coalescence. Peter Thurgood et al. studied the law of vortex generated by microstructures on particle migration and aggregation by adding U-shaped and spherical microstructures in microchannels [29,30]. When the emulsion flows within the microchannel, the hydraulic equilibrium is used to concentrate the droplets in a certain area, thereby increasing the probability of aggregation between the droplets [31,32,33,34].

The 3D-ESPM employs a special spiral inner microstructure and a DC electric field at the same time, and also shows an efficient demulsification action on W/O emulsions [16]; however, the demulsifying process and the mechanism still deserve to be studied furthermore, especially, how the induction period affects the demulsification process under the dual coupling action of electric field and hydrodynamic force in the microchannel, what is the cut size of this technology and problems like that have not been reported up to now. Therefore, the demulsification process of W/O emulsions with 3D-ESPM was further investigated and are discussed in this paper in-depth.

## 2. Experimental Section

### 2.1. Materials

Kerosene was purchased from China Petrochemical Group Co., Ltd. (Chengdu, China); Sulfuric Acid, Span80 and Anhydrous Ethanol were all purchased from Kelong Chemicals Co., Ltd. (Chengdu, China). Sulfuric Acid, Span80 and Anhydrous Ethanol were all of the analytical grades and used as received.

### 2.2. Experimental Setup

**Figure 1:**
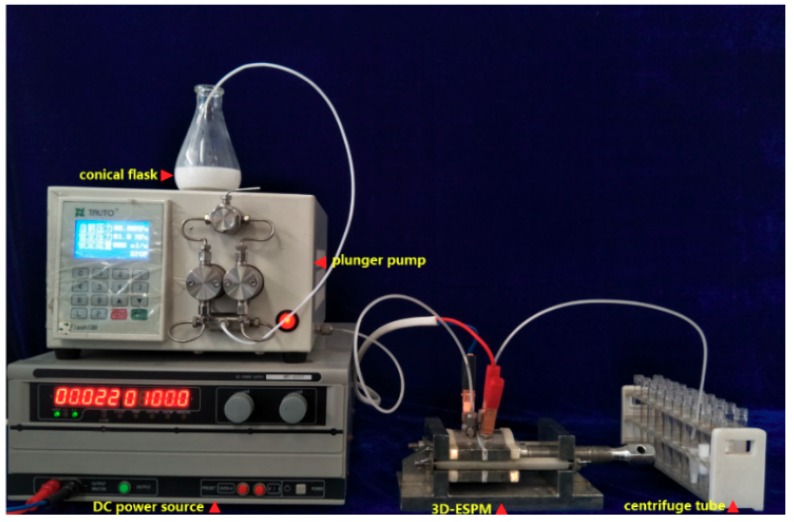
Photograph of the three-dimensional electric spiral plate-type microchannel (3D-ESPM) experimental setup.

The experimental apparatus is shown in Figure 1, which is composed of a conical flask, a medium pressure piston pump (Flash100, TongTian biotech, Shanghai, China), a direct current (DC) stabilized voltage power supply (MWY-40010, Zhongzheng Equipment, Shenzheng, China), three-dimensional electric spiral plate-type microchannel (3D-ESPM) and effluent collection tubes.

**Figure 2:**
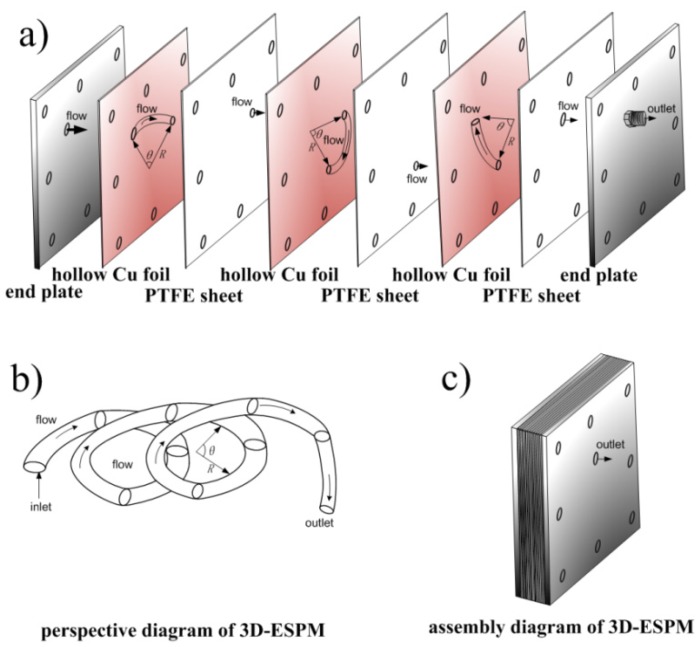
Schematic diagram of the 3D-ESPM. (**a**) Assembly diagram, (**b**) perspective diagram, (**c**) assembled diagram.

The structure of 3D-ESPM was shown in Figure 2. The microchannel was composed of an alternating arrangement of hollow copper foils (200 μm, Golden Dragon, Chengdu, China) and polytetrafluoroethylene (PTFE) sheets (100 μm, Resistance Sealing Material, Chengdu, China), and two stainless steel end-plates. A segmented arch with radian of θ was wire-electrode etched onto each hollow Cu foil, and one orifice corresponding to the end of the arc was pierced onto the PTFE sheets. At both ends along the arrangement line, there were two pieces of stainless steel end-plates, which were 5 mm in depth and with the orifices of entrance and exit. When the apparatus was assembled as structured and as shown in Figure 2a, a spiral microchannel was built along the arch between the PTFE sheets, as shown in Figure 2b, and the whole diagram of the spiral microchannel was shown in Figure 2c. The fabrication details of the 3D-ESPM device can refer to our previous article [16].

### 2.3. Preparation of the W/O Emulsion

The method of preparing the target emulsion [16] was as follows: Firstly, the sulfuric acid (H_2_SO_4_) solution (0.25 mol/L) and the Span80 emulsifier (100 g/L) were pre-prepared. Secondly, 3 mL Span80 emulsifier, 47 mL kerosene and 40 mL H_2_SO_4_ solution will be added in sequence and then mixed. Thirdly, the mixed solution was stirred for two min at 14,000 RPM speed. After settling for 2 min, the stability of emulsion was evaluated according to Equation (1):

(1) $$J=\frac{V_{\mathrm{emulsion}}}{V_{\mathrm{total}}}=\frac{V_{\mathrm{total}}-V_{\mathrm{water}}-V_{\mathrm{kerosene}}}{V_{\mathrm{total}}}$$

where *J* is the stability of the emulsion, *V*_emulsion_ is the volume of the static residual emulsion; *V*_total_ is the total volume of the collected emulsion before the settlement; *V*_water_ is the volume of the separated water in the emulsion; *V*_kerosene_ is the volume of separated kerosene in the oil phase. The emulsion itself is a thermodynamically unstable system, and the stability of the emulsion decreases over time [35,36]. The stability of the target emulsion is able to reach over 88% in 2 h.

### 2.4. Demulsification Experiment

#### 2.4.1. Three-Dimensional Electric Spiral Plate-Type Microchannel (3D-ESPM) Demulsification Experiment

Firstly, the prepared target emulsion was sent into the three-dimensional microchannel by using a medium pressure piston pump, while the DC power supply was used to apply the electric field of the required rate in the microchannel ends. Thus, the emulsion was demulsified in the microchannel. Finally, the tail liquid after the demulsification was collected. The result was recorded to calculate the demulsification rate according to Equation (2):

(2) $$\eta =\frac{V_{\mathrm{water}}{}^{\prime }}{V_{\mathrm{total}}{}^{\prime }\cdot \varphi }\times 100\%$$

where, *η* is the emulsion of the demulsification rate, *V*_water_′ is the volume of water separating out after demulsification, *V*_total_′ is the total volume of the collected liquid from the tail liquid tube, *φ* is the rate of water content in the emulsion.

#### 2.4.2. The Control Experiment

In order to explore the role of the spiral plate microchannel, we replaced the 3D-ESPM with a straight PTFE tube with a diameter of 3 mm and a length of 30 cm, and placed it in an electric field with the strength of 400 V/cm. The control was experimented by pumping the target emulsion through the PTFE tube and keeping the other experimental parameters the same as 2.4.1.

## 3. Results and Discussion

### 3.1. Contrasts between the Gravity Settling and the 3D-ESPM

**Figure 3:**
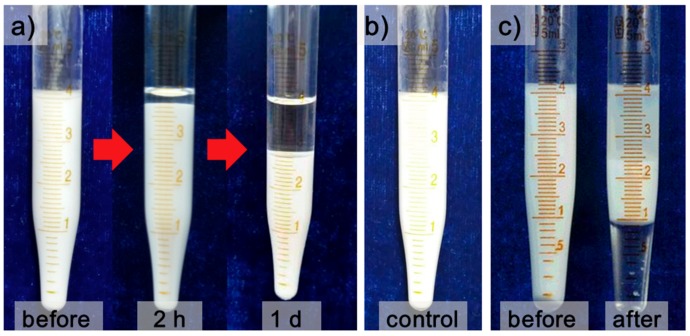
(**a**) Emulsion settling under gravity after 2 h and 24 h, (**b**) emulsion after passing a straight tube in an electric field and (**c**) emulsion before and after a single-pass demulsification with the 3D-ESPM.

One of two samples of freshly prepared water in kerosene emulsions was settled with gravity for 24 h, and the other one was pumped through 3D-ESPM directly with an electric field intensity of 250 V/cm at the same time. Figure 3a,b show the pictures of emulsions demulsified with gravity settling and 3D-ESPM, respectively.

In Figure 3a, only a layer of 0.3 mL oil phase, which is the continuous phase with low density, can be separated from 4 mL W/O emulsion after 24 h of gravity sediment, which indicates that the gravity can hardly separate the dispersed phase water from this W/O emulsion. In addition, we also carried out a control experiment, the results of which are shown in Figure 3b. Obviously, there was no demulsification after the emulsion passed through a straight tube placed at an electric field strength of 250 V/cm. By contrast, in Figure 3c nearly 1 mL clear water phase and 1.8 mL upper oil phase were isolated from 4.3 mL W/O emulsion in 0.7–3 s after emulsion demulsified by 3D-ESPM in a single pass, which indicates that the 3D-ESPM can directly separate the dispersed water droplets and achieve a rapid and effective demulsification process.

From Figure 3, it is clear that only a small amount of oil was stratified on the emulsion surface under gravity sedimentation. However, after having been separated by the 3D-ESPM, a clear water phase and a translucent oil phase were stratified at the bottom and on the surface of the emulsion, respectively. The water-in-kerosene emulsion contains the emulsifier of Span80, which can stabilize the water droplets and prevent the aggregation of droplets from occurring. Therefore, when the emulsion was settled by gravity, the separation comes mostly from the sinking process of water droplets due to the distinct higher density, which will get a clear oil phase on the surface. However, after the gravity settling, the water droplets were still stable and not broken into a continuous phase. Contrary to this, the separation in 3D-ESPM was targeted on the water droplets of emulsions. Under the Dean-coupled inertial migration principle from the spiral microchannel [37,38] and the electrostatic forces from the DC electric field [39], different particle sizes resulting in different movement speeds, the droplets are brought close to each other and are coalesced into an aqueous phase with a higher probability. Thus, the clear water phase will appear at the bottom immediately after the emulsion passes through the 3D-ESPM in 3 s. From our previous report, it has been confirmed that only the DC electric field or only the spiral microchannel did not show obvious demulsification efficiency [16]. However, by comparing the different demulsification effects of gravity settling and 3D-ESPM in Figure 3, we conclude that the DC field can act directly on the charged droplets dispersed in the emulsion and couple with the surface action and microfluidic interaction in the microchannel to enhance the separation of the W/O emulsion. A maximum 40.1% v/v clear water on the bottom, and 50.2% v/v oil layer on the top after emulsion demulsified by 3D-ESPM in 2.4 s, indicated that the coupling action of the microchannel and DC field can efficiently separate the water droplets in the W/O emulsions, by coalescing them into big droplets and generating the phase separation.

**Figure 4:**
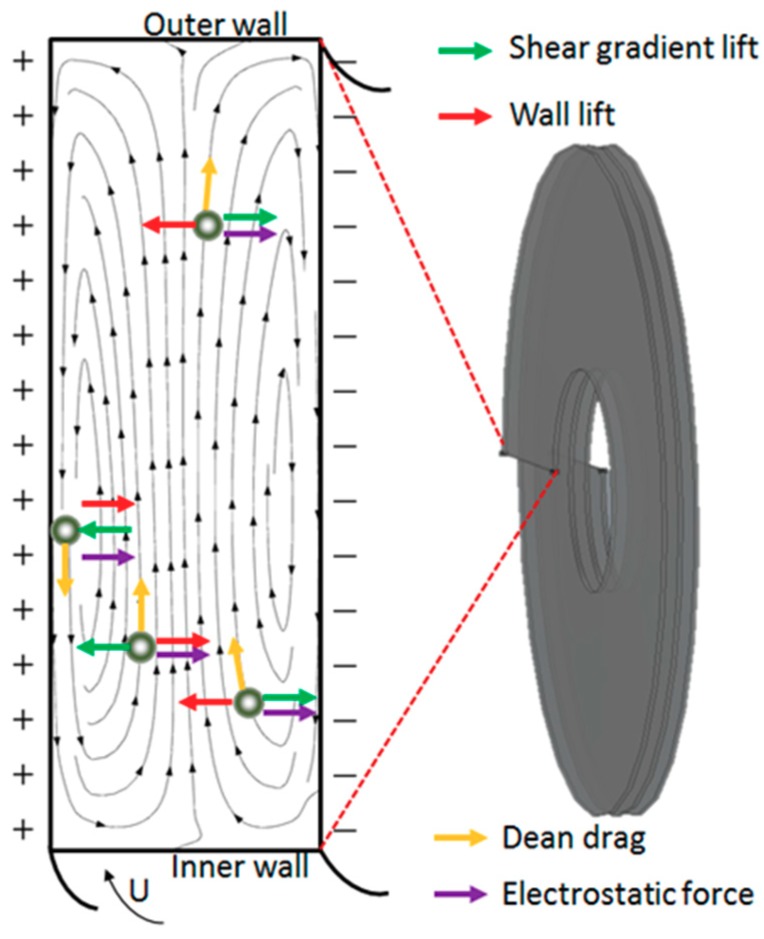
Schematics showing on the droplets at different locations in the 3D-ESPM.

We have analyzed the principle of electric field and hydrodynamic forces in this device and the results were shown in Figure 4. In 3D-ESPM, the droplets are mainly subjected to electrostatic forces, wall lift, viscous drag, shear gradient lift and Dean drag [29,30]. Both the wall lift and the shear gradient lift are hydrodynamic forces, which lead the water droplets moving along the axial direction of the channel up and down migration. The electrostatic forces push the droplets to move in the direction of the electric field, that is, the vertical channel direction. Whereas, the Dean drag, which belongs to the secondary flows, generates the upper and lower vortices in the cross-sectional direction of the channel, which pushes the water droplets to generate double eddy current disturbance in the cross-section direction. Finally, the movement of the droplets in the microchannels is a result of the integration of these three types of forces.

As the flow rate increases, the vortices generated in the microchannels increase, causing the droplets to accumulate at equilibrium positions and the aggregation of droplets of different sizes. Different speeds lead to an instability of the emulsion system and increase the probability of droplets gathering. To a certain extent, an increase in flow rate will enhance the effect of droplet coalescence. In order to further verify the demulsification mechanism of 3D-ESPM, the effects of emulsion flow rate and plate number were studied.

### 3.2. Influence of the Flow Rate and Plate Number

**Figure 5:**
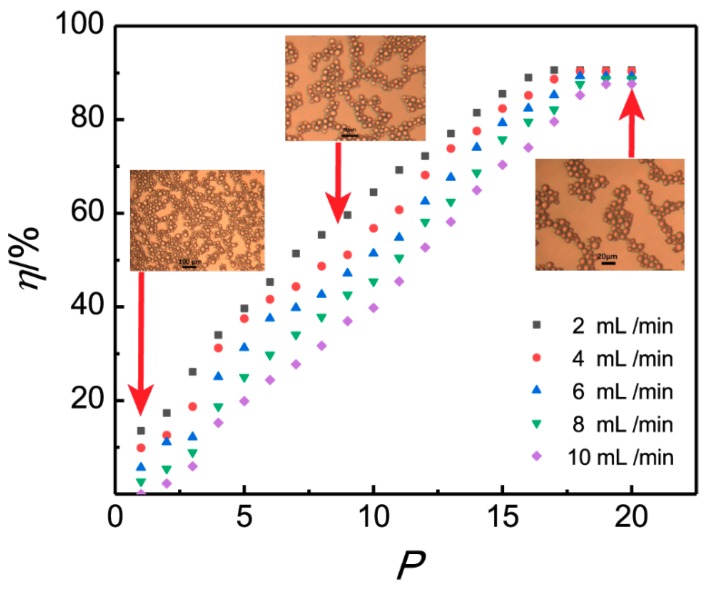
Influence of the plate number on the demulsification efficiency.

Effect of the plate number of 3D-ESPM and the flow rate of W/O emulsion on demulsifying efficiency were studied under the condition of a microchannel height of 200 μm, arc radian of 180° and electric field intensity of 250 V/cm. The plate numbers of 1–20 and flow rates of 2, 4, 6, 8 and 10 mL/min were experimented by the 3D-ESPM in a single-pass demulsification. The results are shown in Figure 5.

Figure 5 shows that the demulsification efficiency *η* increases with the increasing of the plate number *P* for all five flow rates, and the *η-P* scatter plots prolong into an “S” shaped curve of its increasing trend [40]. For flow rates of 6–10 mL /min, it is clear that demulsification efficiency rises lingeringly with the microchannel plate, increasing from one piece to three pieces, and then the demulsification rates increase sharply, and all the rates reach above 80% when the plate number is up to 17. After that, the increased trend obviously turns slow. The percentage of demulsification efficiency peaks at nearly 90.3% on the plate number of 18. Subsequently, the demulsification efficiency plateaus after the plate number exceeds 18. The residence time of the emulsion in the microchannel can be increased by decreasing the flow rate or increasing the plate number of the microchannel. Because the resident time was prolonged, the demulsification efficiency will be increased.

The “S” curve connection between demulsification efficiency and plate number is similar to the S-type growth curve of typical microorganisms [41], therefore it can be deduced that the initial steadily increasing period of demulsification efficiency belongs to the induction period of demulsification of 3D-ESPM with a small plate number. In this period, dispersed phase droplets of W/O emulsion just begin to collect under the electric field; however, due to the short residence time, the demulsification efficiency increases slowly. Sooner after the residence time oversteps the induction period by adding the microchannel plates, the demulsification efficiency begins to grow rapidly. This rapidly increasing period can be considered as the growth period of demulsification by 3D-ESPM. When the number of microchannel plates exceeds 18, the increasing trend will slow down, peaking at 90.3%.

### 3.3. Induction Period

**Figure 6:**
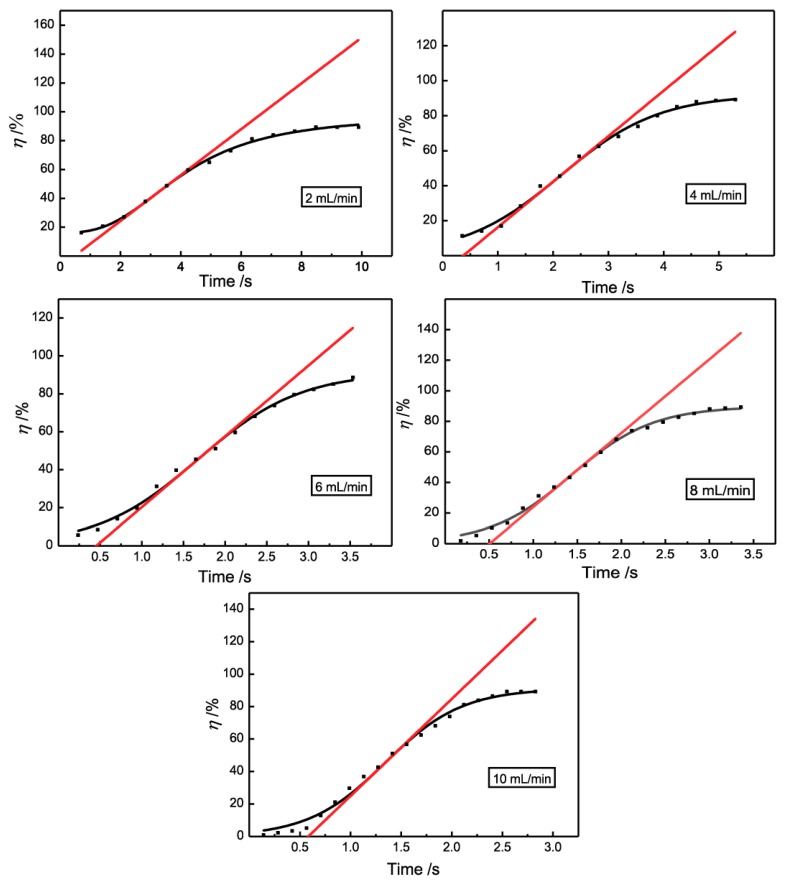
The S-logistic relationship between the de-emulsification efficiency and the resident time.

According to the above results, it is obvious that the demulsification efficiency of 3D-ESPM was influenced by the residence time, and the longer the residence time, the higher the demulsification efficiency. In order to further clarify this relationship, the resident time of emulsions in 3D-ESPM at five flow rates (2, 4, 6, 8 and 10 mL/min) was calculated by varying the plate number, and was plotted with the demulsifcaiton efficiency in Figure 6. In this figure, the experiment results (black dots) were fitted with an S-logistic function (the black spline). Obviously, for all five flow rates, the demulsification efficiencies increase with the rising resident time by following the sigmoidal function congruously, which shows that the S-logistic relationship between *η* and resident time is common for the experimental range of the flow rate. The tangent line at the inflection point of the S-logistic spline was drawn as a red straight line, and for all flow rates the intersection of tangent extension and time axis are positive, which is shown in Figure 6. The inflection point and intercept are shown in Table 1.

Similar to the growth curve of a microorganism, the intercepts in Figure 6 and Table 1 can be considered as the induction period of the demulsification process with 3D-ESPM. Before demulsification and oil-water two phase separation, emulsion droplets are proposed to coalesce by breaking the interfacial film between two droplets. The fast-growing, high-efficiency demulsification process might be due to coalescence propagation [42,43]. Obviously, the induction period is the pre-demulsified process for droplets to aggregate and coalesce before the fast-growing, high-efficiency demulsification process. As shown in Table 1, for the demulsification process with 3D-ESPM, the induction period is in a narrow range from 0.39 to 0.58 s, which demonstrates the high efficiency of 3D-ESPM on coalescing water droplets of W/O emulsions. These are integrate results of electric fields and microchannels.

### 3.4. Influence of the Residence Time

**Figure 7:**
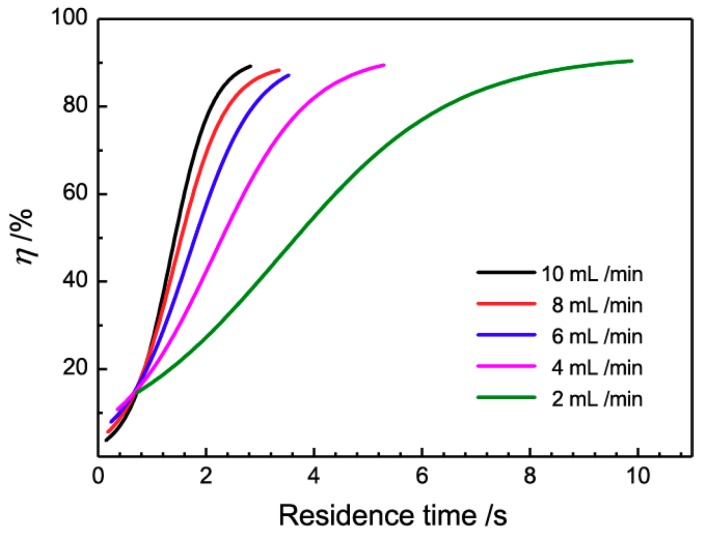
Influence of residence time on the demulsification efficiency.

In order to clarify the influence of the residence time on the demulsifying efficiency, the five data curves of Figure 6 were combined in Figure 7.

From Figure 7, it is obviously that demulsifying efficiencies increase with the increasing residence time for all five flow rates. Besides this, a special phenomenon is that the intersection points of five curves converge on one point at the residence time of 0.6 s, close to the induction period. Below this point, the demulsifying efficiency at a low flow rate is higher than that at a fast flow rate with the same residence time; this may due to the low shear force at the low flow rate, which is in accordance with the anticipation that droplets coalesce easier at this low flow rate. However, beyond this point, this relationship is reversed, and the demulsifying efficiency at the fast flow rate increases sharply and exceeds that at the low flow rate. This phenomenon suggests an extra demulsification impetus in the 3D-ESPM at a fast flow rate. It is reminiscent of the theory that Dean vortices induced droplet coalescence in the spiral microchannels at the high flow rate [44,45,46]. The intensity of Dean vortices is determined by the Dean number *De* according to Equations (3) and (4).

(3) De=Redidc

(4) $$Re=\frac{\rho vdi}{\mu }$$

where *d_i_* is the hydraulic equivalent diameter of the microchannel, *d_c_* is the spiral diameter, *Re* is the Reynolds number, *ρ* is the density of emulsion, *v* is the flow rate of emulsion, and *µ* is the dynamic viscosity of the emulsion.

From the Equations (3) and (4), the Dean number is proportional to the flow rate of the emulsion. The number of this Dean number is 1.80–9.02 when the flow rate is 2–10 mL/min. With the increase of the emulsion flow rate, the Dean vortices in the 3D-ESPM gradually form and strengthen the coalescence of droplets in the emulsion fluid which can intensify the demulsification process. The above experimental results show that the Dean vortex dominates the demulsification process of the 3D-ESPM when the residence time is longer than the induction period.

### 3.5. Droplet size Distributions of Emulsions

**Figure 8:**
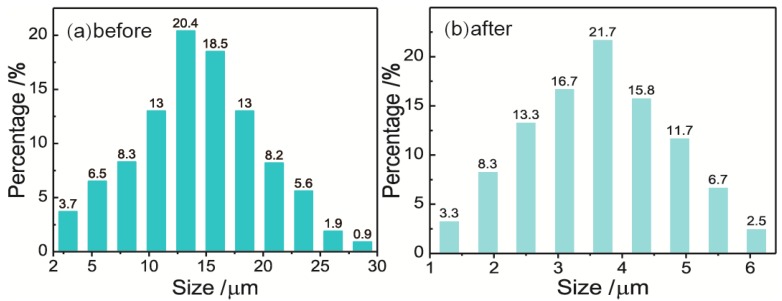
Droplet size distributions of water in oil (W/O) emulsions before (**a**) and after (**b**) having been demulsified by 3D-ESPM. Microchannel height: 200 μm, arc radian: 180°, electric field intensity: 250 V/cm, plate number: 18 and flow rate: 2 mL/min.

Droplet size distributions of emulsions before and after becoming demulsified with 3D-ESPM were measured by using an optical microscope (UB200i, UOP Photoelectric Technology, Chongqing, China) with an image processing software. The results are shown in Figure 8.

As shown in Figure 8, both droplet size distributions of the W/O emulsions before and after being demulsified are in the Normal distribution, however, the distribution range shrinks from 1–30 μm (Figure 8a) to 1–6.5 μm (Figure 8b) after being demulsified. Correspondingly, the average droplet diameter of the emulsions, *D*_(4,3)_, decreases from 19.34 μm to 4.54 μm after demulsification by 3D-ESPM with a demulsifying efficiency of 90.3%. This result indicated that 3D-ESPM preferentially separates the large droplets with a diameter of more than 7 μm, which coalesce to form a water phase, and are absent in the droplet size distribution after having been demulsified. For the small droplets with a diameter of fewer than 7 μm, the percentage of droplets with a diameter from 3.5 to 6 μm in Figure 8a obviously decreases in Figure 8b, which means that 3D-ESPM can also separate droplets with a diameter within this range. Especially from Figure 8a, we can calculate that the volume percentage of the droplets with a diameter above 4.5 μm is 90%, which coincides with the demulsification rate of 90.3%. Therefore, it can be deduced that the cut size of the 3D-ESPM to W/O emulsion is about 4.5 μm, and as a contrast, the cut size of hydrocyclone separation technology is about 10–40 μm [47,48], which reflects that the former can demulsify dispersed droplets with a wider size range than the latter.

## 4. Conclusions

The rapid and efficient separation of the dispersed phase (water) in W/O emulsions was achieved in 0.6–10 s by flowing through the 3D-ESPM under an electric field. Because of the dual coupling effect of the electric field and the microchannel, the demulsification rate reached the highest percentage, 90.3%, by increasing the residence time (as increasing the number of micro-channel plates). The experimental results show that, under the influence of the Dean vortex, 3D-ESPM can effectively intensify the demulsification process of water in oil emulsions.

## Figures and Tables

**Table 1:** The inflection point and intercept of the S-logistic line between the de-emulsification efficiency and the resident time.

*v*/(mL/min)	Inflection Point/s	Intercept/s
2	3.12	0.50
4	2.15	0.39
6	1.67	0.46
8	1.43	0.50
10	1.33	0.58

## List of Symbol

**Symbol**	**Physical Significance**
*J*	The stability of emulsions
*V*_emulsion_	the volume of static residual emulsion
*V*_total_	the total volume of the collected emulsion before the settlement
*V*_water_	the volume of the separated water in the emulsion
*η*	the emulsion of the demulsification rate
*φ*	the rate of water content in the emulsion
*V*_kerosenel_	the volume of separated kerosene in the oil phase
*V*_total_′	the total volume of the collected liquid
*V*_water_′	the volume of separated water after demulsification
*De*	Dean number
*d_c_*	the spiral diameter
*d_i_*	the hydraulic equivalent diameter of the microchannel
*Re*	the Reynolds number
*ρ*	the density of emulsions
*v*	the flow rate of emulsions
*µ*	the dynamic viscosity of emulsions
*D*_(4,3)_	the mean volume diameter of emulsions
